# PFAS Exposure, Mental Health, and Environmental Justice in the United States: Impacts on Marginalized Communities

**DOI:** 10.3390/ijerph22071116

**Published:** 2025-07-15

**Authors:** Shiryn D. Sukhram, Ji Kim, Sabrina Musovic, Ayotunde Anidugbe, Emiliano Corte, Tasneem Ahsan, Selvia Rofail, Nicolli Mesquita, Miguel Padilla

**Affiliations:** 1 Department of Biology, College of Staten Island, New York, NY 10314, USA; sabrina.musovic@cix.csi.cuny.edu (S.M.); ayotunde.anidugbe@my.liu.edu (A.A.); tasneem.ahsan@cix.csi.cuny.edu (T.A.); selvia.rofail@cix.csi.cuny.edu (S.R.); miguelandrei.padilla@cix.csi.cuny.edu (M.P.); 2 Department of Science and Mathematics, Guttman Community College, New York, NY 10018, USA; emiliano.corte82@guttmanmail.cuny.edu (E.C.); nicolli.mesquita60@guttmanmail.cuny.edu (N.M.)

**Keywords:** environmental justice, marginalized communities, mental health, PFAS, race, social inequalities, water contamination

## Abstract

Per- and polyfluoroalkyl substances (PFASs), commonly known as “forever chemicals”, are synthetic compounds with highly stable carbon–fluorine bonds, making them resistant to environmental degradation. These chemicals accumulate in ecosystems and water supplies, posing significant risks to human health, including cancer, immune system dysfunction, and neurological disorders. However, the mental health impacts of PFAS exposure remain underexplored, particularly in marginalized communities. This review examines the emerging evidence linking PFAS exposure to mental health issues such as anxiety, depression, and cognitive decline, with a focus on communities of color who face heightened vulnerability due to environmental and social health disparities. The review highlights the persistence of PFASs in the environment, common exposure pathways, and the disproportionate effects on populations living near contaminated sites. Despite some regulatory progress, U.S. regulations on PFASs are limited, especially compared to international standards. The review calls for stronger policy frameworks and emphasizes the need for environmental justice, health equity, and public awareness. By connecting environmental health, social justice, and mental well-being, the review aims to guide future research and policy reforms to mitigate the mental health consequences of PFAS exposure in vulnerable populations.

## 1. Introduction

Per- and polyfluoroalkyl substances (PFASs) are a class of synthetic chemicals that have garnered significant attention due to their environmental persistence and potential adverse health effects [1,2,3,4,5,6,7]. Often referred to as “forever chemicals”, PFASs are used in a wide range of industrial and consumer products, including nonstick cookware, water-repellent clothing, and aqueous film-forming foam (AFFF). These compounds resist degradation, leading to widespread accumulation in ecosystems [3,4,8,9,10,11].

Despite growing public concern, substantial knowledge gaps remain regarding the full spectrum of PFAS-related health effects—particularly those related to mental health and the disproportionate burden on marginalized communities [12,13,14]. This review aims to critically examine the intersection between PFAS exposure and mental health, with a particular emphasis on communities of color. While the existing literature [4,15,16,17,18] has extensively documented PFAS’ physical health impacts—such as cancer, immune dysfunction, and reproductive harm—their potential effects on mental health, including anxiety, depression, and cognitive impairments, have only recently garnered attention. Moreover, the environmental and social determinants contributing to heightened exposure among marginalized populations remain underexplored.

The first section of this review provides an overview of PFASs, including their chemical properties, sources of exposure, and environmental persistence. PFASs are now ubiquitous in the environment, particularly in drinking water, and have been shown to bioaccumulate in human and animal tissues. Exposure occurs through various pathways, including industrial emissions, contaminated water supplies, and food sources. The global distribution and persistence of these compounds draw attention to their widespread impact on ecosystems and human health.

Subsequent sections focus on the health effects of PFASs, with a particular emphasis on emerging research related to mental health. We review findings linking PFAS exposure to a range of adverse outcomes, including carcinogenicity, immune system disruption, and endocrine interference, while also highlighting new evidence on mental health consequences. Bioaccumulation in tissues such as blood will be discussed in the context of chronic exposure and long-term health implications.

A central theme of this review is the disproportionate exposure burden borne by marginalized communities—particularly communities of color. These populations often reside near contaminated sites, face barriers to healthcare access, and experience systemic inequities in environmental regulation [12,19,20,21,22,23]. We examine case studies that illustrate these disparities and their implications for mental health, highlighting PFAS exposure as a pressing environmental justice issue.

Finally, we assess the current policy landscape governing PFAS regulation, identifying critical gaps and proposing recommendations to strengthen environmental protections. Special attention is given to the need for policy frameworks that prioritize equity and provide adequate support to the most affected communities.

This review adopts a holistic approach that integrates environmental health, mental well-being, and social justice. By focusing on the intersectionality of PFAS contamination and mental health in communities of color, we aim to advance the understanding of this urgent public health issue and contribute to broader efforts to achieve environmental health equity. Through this synthesis, we seek to inform policy, raise public awareness, and support evidence-based action to mitigate PFAS-related risks.

## 2. PFAS: Chemistry, Sources, and Environmental Persistence

### 2.1. Chemical Properties of PFAS

**Figure 1:**
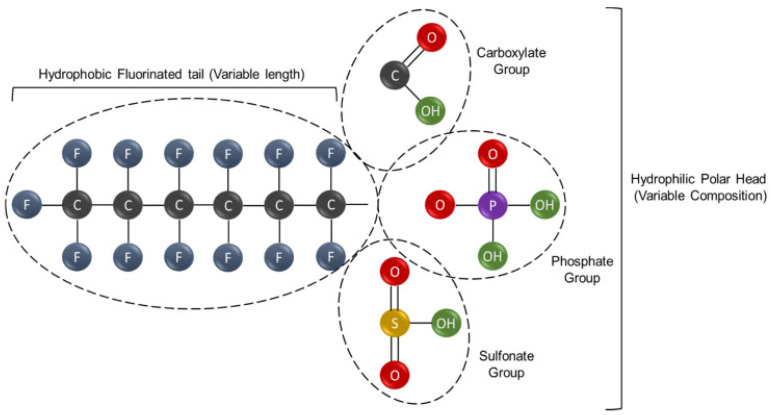
General structure of a fluorinated compound showing a hydrophobic fluorinated tail (**left**) and a hydrophilic polar head (**right**) with variable functional groups: carboxylate, phosphate, and sulfonate. Atom colors: C (black), F (blue), O (red), H (green), P (purple), and S (yellow). Adapted from Panieri et al. [5].

PFASs are a class of environmentally persistent pollutants that exhibit significant structural diversity. They are characterized by carbon chains that are either partially (poly-) or fully (per-)fluorinated [5,24]. A defining feature of PFASs is their amphiphilic nature, as displayed in Figure 1, arising from a fluorinated alkyl tail that displays both hydrophobic and hydrophilic properties [4,5,17]. The carbon–fluorine bond—among the strongest and shortest in organic chemistry—confers exceptional stability, making these compounds highly resistant to environmental degradation. Thus, the chemical stability of PFASs is the basis for their classification as “forever chemicals” and contributes to their accumulation in various human and wildlife tissues [25,26,27,28]. These compounds have been detected in multiple human biological matrices, including blood serum, the liver, kidneys, and brain tissue. Their strong binding affinity for proteins such as albumin enables widespread distribution within the body. Evidence of PFAS presence in umbilical cord blood, placenta, and breast milk demonstrates maternal transfer during gestation and lactation. In wildlife, PFAS accumulation is commonly observed in the liver, blood, and adipose tissue, as well as in aquatic organisms like fish, where they bioaccumulate and biomagnify through trophic levels. The persistence of PFASs within these tissues raises significant concerns regarding chronic exposure and potential cumulative toxicity across generations [28,29,30,31].

**Figure 2:**
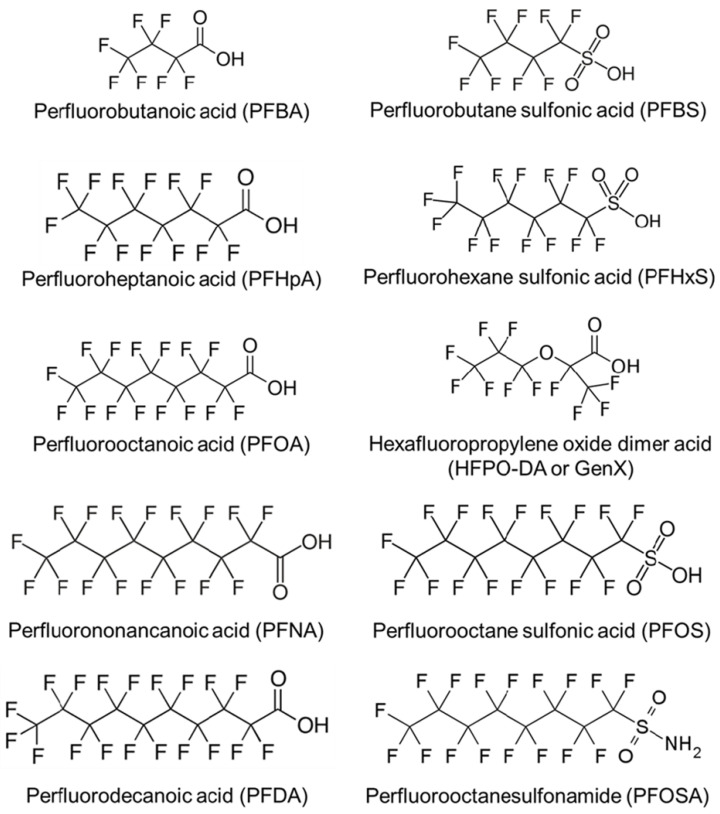
Structural diversity of key per- and polyfluoroalkyl substances implicated in environmental persistence and human exposure. Adapted from Blake & Fenton [16].

The classification of PFASs is continually evolving as the chemical industry modifies their structures to optimize functional applications. This classification varies across regulatory bodies [32,33,34] depending on their operational criteria and the intended scope and purpose of the chemical inventories they adopt. There are thousands of types of PFASs, and the United States (U.S.) Environmental Protection Agency (EPA) [32] has identified several PFASs of concern due to their persistence, bioaccumulation potential, and associated health risks. Figure 2 illustrates the molecular structures of some, which are among the most widely studied [16]. Perfluorooctanoic acid (PFOA) and perfluorooctane sulfonic acid (PFOS), once widely used in products like nonstick cookware, carpets, and AFFF, are no longer manufactured domestically and are classified as “carcinogenic” according to the International Agency for Research on Cancer (IARC) [15,35]. GenX^®^ chemicals, such as hexafluoropropylene oxide dimer acid (HFPO-DA), were developed as replacements for PFOA but are more environmentally mobile and have been associated with a range of adverse effects, including hepatotoxicity, reproductive and developmental toxicity, neurotoxicity, intestinal and immunotoxicity, hematological and renal effects, as well as carcinogenic and genotoxic potential [36,37,38,39]. Perfluorobutane sulfonate (PFBS) and perfluorooctanoic acid (PFNA) are used in textiles, food packaging, and cleaning agents, with emerging evidence linking them to thyroid disruption and reproductive toxicity, respectively [30,40,41]. Perfluorohexane sulfonate (PFHxS), which remains present in some imported goods, has been associated with adverse effects on the liver and immune system [42,43]. Perfluorodecanoic acid (PFDA), a long-chain PFAS used in industrial applications, bioaccumulates and may impair endocrine and reproductive function [6,16,18]. Perfluorobutanoic acid (PFBA), used in cleaning products and film, shows low bioaccumulation but potential respiratory and renal toxicity [44]. Lastly, perfluoroheptanoic acid (PFHpA), structurally similar to PFOA, has been detected in drinking water and may pose developmental and immune health risks [45,46]. Collectively, these PFASs raise significant regulatory and public health concerns, prompting federal limits on their presence in the environment [47].

PFOA and PFOS are the most extensively studied, largely due to their role as major byproducts of fluoropolymer manufacturing [48,49,50]. For the purposes of this review, long-chain PFASs are defined as compounds with seven or more carbon atoms, while short-chain PFASs contain six or fewer. Long-chain PFASs are more prevalent in environmental and human samples and are associated with more severe adverse health outcomes [29,50,51].

As regulatory efforts increasingly target long-chain PFASs, scientific attention has shifted toward evaluating the potential health risks associated with their short-chain alternatives. While the evidence remains inconclusive regarding the influence of carbon chain length on bioaccumulation, emerging studies [5,17,52,53] suggest that certain short-chain PFASs may exhibit environmental persistence and toxicological profiles comparable to those of long-chain compounds. This remains an active area of investigation, particularly with regard to the comparative neurotoxicological effects of both PFAS classes. These substances can accumulate in the human body over time, as the strength of the carbon–fluorine bonds renders them highly resistant to metabolic degradation. Although the precise biological retention times are still being characterized, current evidence indicates that PFASs may persist in human tissues for decades [25,29,54].

### 2.2. Sources of Exposure

PFASs are commonly found in household cleaning agents, nonstick cookware, grease-resistant food packaging, and drinking water [26,55]. PFASs are also present in water-repellent textiles such as rain jackets, umbrellas, and tents, as well as in stain-resistant coatings applied to carpets, upholstery, and other fabrics [33]. Additionally, personal care products, including shampoo, dental floss, nail polish, and eye makeup, may contain PFASs, contributing to widespread human exposure through everyday use [34]. The U.S. Food and Drug Administration (FDA) [56] has also identified PFASs in various cosmetic and personal care products. However, the extent to which PFASs can be absorbed through the skin from cosmetic use remains unclear and warrants further investigation. The Modernization of Cosmetics Regulation Act of 2022 (MoCRA) represents the most substantial enhancement of the FDA’s regulatory authority over cosmetics since the enactment of the Federal Food, Drug, and Cosmetic Act of 1938. Under MoCRA, the FDA is mandated to evaluate the use and safety of PFASs in cosmetic products—potentially in consultation with the National Center for Toxicological Research—and to publish a summary report of its findings by 29 December 2025. Although regulatory measures have since restricted the use of PFASs in numerous consumer products, their environmental persistence indicates that contamination will continue to pose a significant public health risk well into the future [5,57].

The widespread use of PFASs has significantly contributed to environmental contamination, particularly in drinking water sources. A national pilot study conducted by Smalling et al. [38] analyzed 716 tap water samples collected from residential, commercial, and drinking water treatment locations throughout the U.S. from 2016 to 2021. In 2021, 409 point-of-use samples were collected from 155 private wells and 252 public water systems spanning all 50 states, the District of Columbia, Puerto Rico, and the U.S. Virgin Islands, utilizing a volunteer-based sampling network. The modeling results indicated the presence of at least one PFAS compound in 45% of U.S. drinking water samples, with several samples from both private wells and public water supplies exceeding proposed federal PFAS regulatory limits and health benchmarks.

One notable case of PFAS water contamination occurred in Newburgh, New York (NY), where PFOS, PFOA, and PFHxS were first detected in 2013. In response, the city switched to an alternative water source by 2016, significantly reducing PFAS levels [38]. Nevertheless, contamination has since spread to other areas, especially the Hudson Valley, now considered a hotspot for PFOS and PFOA [30,58]. In the absence of comprehensive federal regulation, NY State has designated PFOS and PFOA as hazardous substances and initiated a statewide effort to remediate contaminated sites [59].

Occupational exposure to PFASs often differs substantially from that of the general population, as workers may be exposed to concentrated PFAS-containing substances through dermal contact or inhalation of airborne particles in occupational settings [60,61]. The extent and nature of this exposure depend on factors such as industry type, specific job functions, and workplace tasks. Elevated levels of PFASs have been documented among workers in industries such as chemical manufacturing, metal plating, firefighting, and ski waxing, compared to the broader U.S. population [62,63]. Given the pervasive nature and environmental persistence of PFASs, this represents a pressing public health challenge.

### 2.3. Environmental Accumulation

PFASs comprise a large class of thousands of synthetic chemicals that have been used globally in industrial applications and consumer products for several decades [25,64]. These substances enter the environment through various pathways, including discharges from landfills, wastewater treatment plants, and industrial facilities, as well as runoff from military installations and areas treated with AFFF or sewage sludge [31,65]. Industries such as paper and textile manufacturing, along with facilities producing AFFF, contribute substantially to PFAS emissions, particularly when sewage sludge is improperly managed [66]. Military activities at air bases, airports, and training grounds further exacerbate groundwater contamination through waste disposal, equipment testing, leaks, and fire suppression practices [31,67]. Additional contributors include municipal solid waste leachate, treated effluent, and the agricultural application of biosolids [66]. Once released into groundwater systems, PFASs persist due to their resistance to conventional water treatment technologies, thereby posing ongoing risks to human health and the environment [66,68].

PFAS contamination is now recognized as a global concern, with detections reported in both industrialized and developing regions, including remote locations such as the Arctic and North Atlantic Ocean. This review, however, focuses primarily on the U.S. These chemicals are capable of long-range transport via atmospheric and oceanic currents. Lohmann et al. [69] documented substantial PFAS circulation in the Arctic Ocean, with roughly equal amounts entering and exiting the region, demonstrating their environmental persistence and mobility. Despite regulatory phase-outs of certain long-chain PFASs, newer short-chain variants continue to be detected, including at ocean depths exceeding 3000 feet, raising ongoing concerns about widespread exposure and ecological impact [65,69].

In the U.S., Michigan has emerged as a PFAS contamination hotspot due to its extensive industrial activity, historical waste disposal practices, and the presence of multiple military installations [70]. Wurtsmith Air Force Base (AFB) and Alpena Combat Readiness Training Center have been identified as major sources of contamination, primarily through the extensive use of AFFF during firefighting training and emergency response [70,71,72]. At Wurtsmith AFB in Oscoda, the application of AFFF led to significant PFAS release, with compounds leaching into soil, infiltrating groundwater, and contaminating nearby water bodies such as Van Etten Lake and Clark’s Marsh [72]. Initial testing by the Michigan Department of Environment, Great Lakes, and Energy (EGLE) and the U.S. Air Force confirmed elevated PFAS levels in areas affected by past firefighting activities [73,74]. Subsequent investigations revealed widespread contamination in both on-base and off-base water sources. In response, state officials have recommended the expanded testing of private wells and public water systems in the surrounding area to determine the full extent of exposure [75]. Legal action is currently under consideration for affected individuals, including military personnel, their families, and nearby residents, who were exposed to PFAS-contaminated drinking water and have experienced adverse health effects [76].

Contaminated drinking water remains a critical public health concern, as PFASs infiltrate both public and private water systems. According to the EPA [77], over 158 million Americans face the risk of drinking PFAS-contaminated water, with approximately 9190 sites across the country reporting detections. The Fifth Unregulated Contaminant Monitoring Rule (UCMR 5) [77] reveals that approximately 53 million individuals residing in states without specific PFAS drinking water regulations are exposed to PFAS concentrations exceeding the limits set by the EPA. In the absence of state-level protections, these populations depend entirely on federal standards to mitigate health risks associated with PFAS-contaminated drinking water.

One of the most concerning properties of PFASs is their chemical stability, which enables them to bioaccumulate throughout the food chain [3,8,55,78]. In response to the growing evidence linking specific PFASs to adverse health effects, the FDA [55,78] has prioritized monitoring and evaluating PFAS contamination in the food supply. Since 2019, the agency has enhanced its analytical methods to detect 30 PFAS compounds across diverse food matrices, tested approximately 1300 food samples from the U.S. market, and supported state-level testing of over 400 samples from areas with known contamination. In addition, the FDA has conducted toxicological assessments on PFASs identified in 174 samples, reviewed post-market exposure data, and collaborated with industry to phase out PFAS-based grease-proofing agents—culminating in regulatory actions finalized in 2024 and 2025 [55].

Aquatic organisms absorb PFASs from contaminated water, and these compounds biomagnify through the food chain, ultimately affecting top predators, including humans who consume seafood [8]. A recent study by the FDA [78] identified PFASs in 23 out of 35 food samples analyzed, with 19 of the positive detections occurring in seafood products. Although data on PFAS concentrations in seafood remain limited, these findings suggest that seafood may be particularly vulnerable to environmental PFAS contamination compared to other food categories. Exposure pathways vary across demographic groups and are influenced by factors such as dietary preferences, geography, and socioeconomic conditions [23,79].

## 3. Health Impacts of PFAS Exposure

### 3.1. Serum Concentrations

Mounting evidence indicates that PFASs contribute to a range of serious health outcomes through both direct (e.g., contaminated drinking water) and indirect (e.g., bioaccumulation in the food chain) exposure pathways [8,38,79,80].

The National Academies of Sciences, Engineering, and Medicine [81] recommend that the Centers for Disease Control and Prevention (CDC) revise its clinical guidelines to encourage clinicians to offer PFAS blood testing for individuals at risk of high exposure, such as those with occupational exposure or residing in contaminated areas. If test results indicate elevated PFAS levels associated with increased health risks, patients should be monitored through regular screenings. Furthermore, the CDC, the Agency for Toxic Substances and Disease Registry (ATSDR), and public health departments should support clinicians by providing educational resources on PFAS exposure, potential health effects, testing limitations, and the benefits and drawbacks of testing [81].

Despite growing awareness, the precise toxicological mechanisms of PFASs remain inadequately understood, prompting the use of surveillance tools like the National Health and Nutrition Examination Survey (NHANES) for both quantitative and qualitative exposure assessments [82]. Botelho et al. [83] analyzed trends in PFAS serum concentrations among individuals aged 12 years and older using NHANES data collected from 1999 through March 2020. The analysis confirmed that four legacy PFASs—PFOA, PFOS, PFHxS, and PFNA—remain the most frequently detected compounds in the U.S. population. Since the 1999–2000 survey cycle, over 95% of the participants have shown detectable levels of at least one of these PFASs, with detection rates ranging from 96.86 to 99.77% for PFHxS, 98.97 to 100% for PFOS, 98.94 to 99.99% for PFOA, and 95.00 to 99.81% for PFNA. The study also identified significant associations between demographic factors and PFAS exposure. Specifically, detection of 9-chlorohexadecafluoro-3-oxanonane-1-sulfonic acid (9CLPF), a PFOS replacement compound used in certain industrial applications though not yet widely categorized as a PFAS, was significantly associated with race (Asian vs. non-Asian), country of birth (U.S.-born vs. foreign-born), and duration of residence in the U.S. (<10 years vs. ≥10 years), with all the associations showing statistical significance (Chi-square *p* < 0.01).

PFAS compounds bioaccumulate in human tissues over time and have been detected in both blood and specific regions of the brain [79,84]. Although the exact pathways remain under investigation, current research suggests that fetal exposure may occur via placental transfer [85,86,87,88]. The placenta, which facilitates nutrient and hormone exchange while serving as a protective barrier, is highly permeable to PFASs [88]. As a result, elevated levels of these chemicals have been identified in infants’ blood serum, raising concern about developmental outcomes [86,88]. PFAS exposure during gestation has been linked to disorders such as attention-deficit hyperactivity disorder (ADHD), Down syndrome, and other severe neurological impairments [17].

### 3.2. Bioaccumulation in Brain

**Figure 3:**
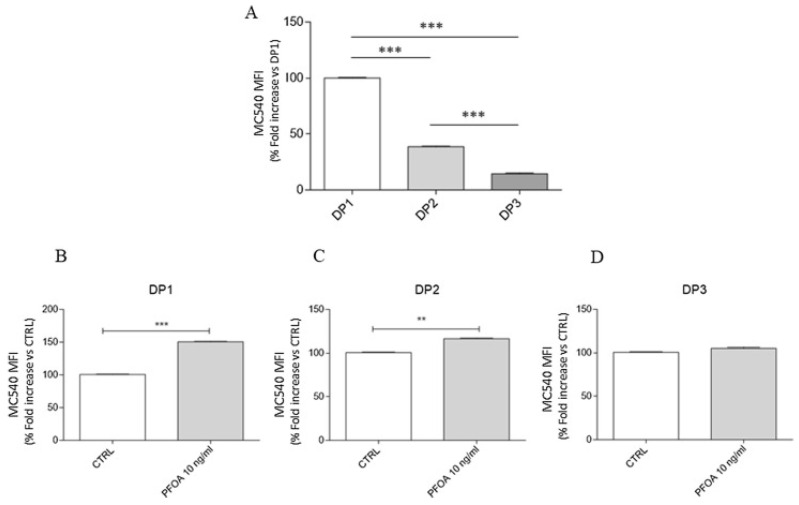
Perfluorooctanoic acid (PFOA)-induced alterations in membrane fluidity across neurodifferentiation phases: commitment (DP1), neuronal precursor (DP2), and mature dopaminergic differentiation (DP3). (**A**) Baseline comparison of MCS40 mean fluorescence intensity (MFI) among differentiation points DP1–DP3, showing a significant decrease in MFI from DP1 to DP3; (**B**) Exposure to PFOA (10 ng/mL) significantly increases MCS40 MFI at DP1 compared to control (CTRL); (**C**) PFOA exposure at DP2 results in a moderate but statistically significant increase in MCS40 MFI; (**D**) No significant difference in MCS40 MFI is observed between control and PFOA-treated cells at DP3. Statistical significance: ** *p* < 0.01, *** *p* < 0.001. Adapted from Di Nisio et al. [84].

Emerging evidence suggests that PFASs may also cross the blood–brain barrier (BBB), potentially contributing to disruptions in brain function [17,84,89,90,91,92,93,94,95,96]. The brainstem, which contains a high density of dopaminergic neurons, has been shown to accumulate significant levels of PFASs in regions with environmental contamination. Di Nisio et al. [84] investigated the effects of PFOA exposure during distinct stages of dopaminergic differentiation—specifically the neuronal commitment phase (DP1), neuronal precursor phase (DP2), and mature dopaminergic differentiation phase (DP3)—by analyzing morphological changes and the expression of key dopaminergic markers using indirect immunofluorescence. Markers such as tyrosine hydroxylase (TH), dopamine transporter (DAT), and neurofilament heavy chain (NF-H) were evaluated to assess the impact of PFOA on dopaminergic development. After 24 h of PFOA exposure at each stage, the following results were observed: at DP1, DAT was undetectable, and NF-H staining was significantly reduced; at DP2, NF-H staining increased, and limited DAT activity was observed; and at DP3, marked downregulation of TH and DAT was detected, with impaired dopaminergic function relative to controls (Figure 3). These findings suggest that PFOA exposure disrupts both early and late dopaminergic differentiation processes, potentially impairing neuronal development and function [84,89,97]. The study also examined factors influencing PFAS absorption, distribution, and accumulation in the brain, identifying correlations between PFAS exposure and altered neurochemical, neurophysiological, and behavioral outcomes [84]. This evidence points to the potential for PFASs, particularly during key stages of neural development, to interfere with the formation and functionality of dopaminergic systems.

The BBB regulates the entry of substances into the central nervous system (CNS) via two main mechanisms: passive transmembrane diffusion and saturable active transport [17]. While transmembrane diffusion permits the passive passage of small molecules, active transport systems involve ATP-dependent processes and ligand-specific interactions [17,98]. The developing brain is particularly susceptible to chemical exposure, owing to the immature state of the BBB during infancy and childhood [99]. This immaturity is characterized by underdeveloped tight junctions, elevated transcytosis, and the reduced expression of efflux transporters compared to the adult BBB [17,99]. Albumin, the primary PFAS-binding protein in circulation, facilitates the systemic distribution and accumulation of these compounds [17,100]. In neonates, the incomplete development of the BBB allows PFASs bound to albumin to more readily penetrate brain tissue [17,101]. Moreover, individuals with autoimmune conditions such as lupus or rheumatoid arthritis may experience compromised BBB integrity, further increasing vulnerability to xenobiotic infiltration, such as PFAS [17]. These combined factors amplify the risk of PFAS-induced neurological damage in sensitive populations such as infants and immunocompromised individuals.

### 3.3. Underexplored Mental Health Effects

**Figure 4:**
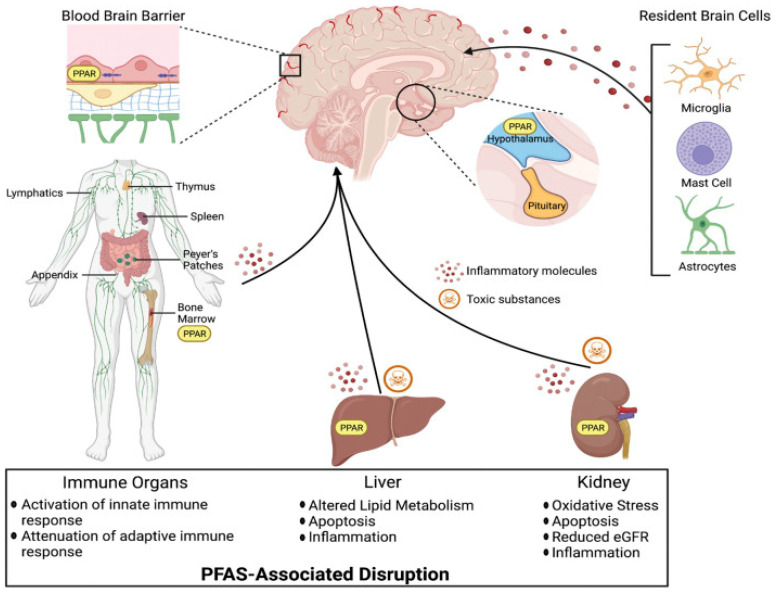
Per- and polyfluoroalkyl substances disrupt brain, immune, liver, and kidney function via peroxisome proliferator-activated receptor (PPAR) activation and inflammatory pathways. Adapted from Starnes et al. [17].

Despite increasing concerns regarding the environmental and physiological impacts of PFASs, the potential relationship between PFAS exposure and mental health outcomes remains inadequately explored. Relatively few studies have investigated these associations, leaving a significant gap in our understanding of the neuropsychological effects of PFASs. Additionally, there is a notable lack of research examining the impact of PFASs on the nigrostriatal system—particularly the striatum and ventral midbrain—as well as other brain regions potentially involved in PFAS-related neurological dysfunction in humans, as illustrated in Figure 4 [17]. This figure illustrates the widespread systemic effects of PFASs, demonstrating how exposure can disrupt multiple organ systems. Major toxicity pathways include the activation of PPAR (peroxisome proliferator-activated receptor) signaling and the generation of inflammatory and toxic molecules. In the CNS, PFASs can impair the BBB and affect resident cells such as microglia, mast cells, and astrocytes, triggering neuroinflammation. In the immune system, PFAS exposure modifies immune responses by amplifying innate immunity while suppressing adaptive defenses. Liver-related effects include disturbances in lipid metabolism, increased cell death, and inflammation, while kidney damage involves oxidative stress, inflammation, reduced estimated glomerular filtration (eGFR), and apoptosis. These widespread biological disruptions highlight the extensive physiological burden of PFAS exposure. The lack of focused research on neural pathways emphasizes the urgent need for studies aimed at clarifying how PFAS exposure may contribute to adverse mental health outcomes.

**Figure 5:**
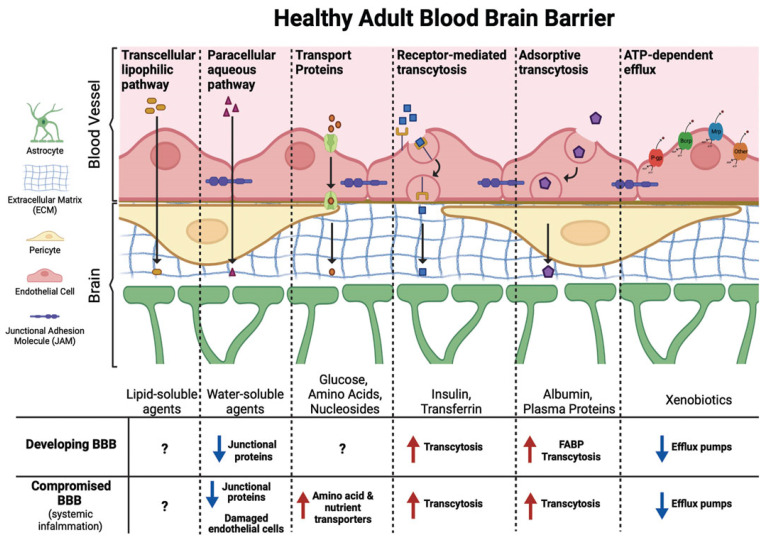
Diverse transport mechanisms across the healthy blood–brain barrier governed by molecular size and biochemical properties. (1) Transcellular lipophilic pathway: Passive diffusion of lipid-soluble molecules across endothelial cells; (2) Paracellular aqueous pathway: Limited diffusion of small water-soluble molecules through tight junctions (blue arrow); (3) Transport proteins: Blue arrows indicate influx transporters that actively move essential nutrients into the brain. Red arrows denote efflux transporters that pump out unwanted substances; (4) Receptor-mediated transcytosis: Blue arrows represent vesicle-mediated uptake of molecules that bind to specific endothelial receptors for targeted delivery into the brain; (5) Adsorptive transcytosis: Blue arrows reflect nonspecific uptake of cationic proteins via charge interactions with the endothelial membrane; (6) ATP-dependent efflux: Red arrows highlight active transporters that use ATP to expel xenobiotics and drugs from the brain, protecting neural tissue. Adapted from Starnes et al. [17].

Starnes et al. [17] revealed a significant increase in glutamate levels in the hippocampus and catecholamine levels in the hypothalamus, alongside a decrease in dopamine levels in the brain following PFAS exposure. The hypothesized mechanism for PFASs crossing the BBB is linked to increased membrane permeability, as illustrated in Figure 5. This disruption may be caused by the PFOS-induced stimulation of reactive oxygen species (ROS) at elevated levels, which leads to the breakdown of tight junction integrity [90]. Furthermore, ADHD has been associated with reductions in dopamine synaptic markers, correlating with symptoms of inattention and distractibility, as observed in the dopamine reward pathway of individuals with ADHD [91]. Several studies suggest a link between PFAS exposure and the manifestation of ADHD symptoms, indicating a potential neurodevelopmental impact of these chemicals [92,93].

Brown-Leung & Cannon [89] propose that PFASs may exhibit neurotoxic properties, potentially contributing to chronic mental health disorders and neurodegenerative diseases. As emerging contaminants, scientists are still in the early stages of understanding the full extent of their effects. While the harmful effects of PFAS exposure are well documented, the potential neurological impacts may be underestimated, in part due to the widespread environmental presence of PFASs and the complexity of attributing specific neurotoxic outcomes to these persistent chemicals [94,95,96].

Mechanistic studies, designed to elucidate the biological processes underlying these phenomena, are critical for advancing our understanding of PFAS neurotoxicity. Future research is essential to comprehensively assess the various exposure routes and examine how PFASs and their mixtures influence neurotransmission mechanisms and related neurobiological effects [96]. Notably, much of the existing research does not disaggregate mental health outcomes by race, which limits our understanding of how environmental stressors like PFASs contribute to mental health disparities.

## 4. Environmental Justice and PFAS

### 4.1. Disproportionate Impact on Marginalized Communities

This review centers on marginalized populations for which a substantial empirical evidence base exists regarding PFAS exposure, primarily including communities of color, low-income groups, and residents living near contaminated sites. Currently, there is a notable scarcity of published case studies examining PFAS exposure among other vulnerable populations, such as individuals with disabilities. We recognize the need for further research to investigate PFAS exposure across a wider spectrum of marginalized groups and highlight this as a critical gap in the existing literature.

PFAS exposure disproportionately affects communities with higher proportions of Black and Hispanic/Latino residents, largely due to their geographic proximity to PFAS-emitting sites such as airports, military bases, and industrial facilities [12,20,102,103,104]. These locations frequently use materials containing PFASs, particularly AFFF [20]. Runoff from these areas facilitates the migration of PFASs into local water supplies, leading to elevated concentrations in drinking water. Low-income urban neighborhoods, especially those with high proportions of racial and ethnic minorities, are frequently situated in close proximity to industrial facilities and waste disposal sites known to release PFASs. For example, communities in Ohio and West Virginia have documented elevated PFAS concentrations in local water sources [105,106]. These populations already experience significant socioeconomic and health disparities, and the added burden of PFAS exposure further compounds these inequities, highlighting critical environmental justice concerns.

Research [12,20,102,103] indicates that Black and Hispanic/Latino communities are 1.5 to 2 times more likely to be exposed to unsafe PFAS levels compared to predominantly white communities. Liddie et al. [20] conducted a multi-state analysis and found that each 1% increase in the proportion of non-Hispanic Black residents in a community was associated with a 6–9% higher likelihood of nearby PFAS-emitting facilities. Similarly, Hispanic populations experienced a 3–6% greater risk. In rural areas, PFAS contamination risk rose by 10% for each additional percent of the population living in poverty [20].

These findings publicize the intersection of environmental injustice and PFAS contamination, where vulnerable communities face heightened exposure due to systemic factors such as location, socioeconomic status (SES), and inadequate regulation. Residents of affected communities often express a need for greater transparency and information regarding PFAS exposure. Calls for biomonitoring, exposure pathway analysis, and health studies reflect the desire to better understand the extent of exposure and identify strategies to mitigate health risks. Biomonitoring involves the measurement of environmental chemicals or their metabolites in human biological specimens (e.g., blood, urine, or breast milk) to directly assess internal exposure levels within populations. These communities, already at increased risk due to discriminatory practices and insufficient protective policies, are seeking actionable information on how to reduce the dangers posed by PFASs. Addressing these concerns through continued research and improved regulatory frameworks is essential to protect public health and reduce environmental disparities.

### 4.2. Systemic Racism in PFAS Exposure Disparities

Libenson et al. [21] reported the disproportionate exposure of communities of color, particularly in California, to PFAS contamination in drinking water. One significant source of this contamination is runoff from agricultural pesticides. When applied near local water supply wells, these pesticides can lead to PFAS infiltration in nearby water systems. This issue is especially prominent in regions with large Latinx and non-Latinx communities of color, where agriculture is prevalent and small, and less-regulated water systems are common. In rural and agricultural areas, such as the San Joaquin Valley, Central Coast, and Inland Empire, PFAS contamination is exacerbated by the widespread use of pesticides [107]. Areas with a higher proportion of Latinx populations—specifically those with at least 10% Latinx residents—experienced a 27% increase in PFAS application density [21]. Additionally, the study noted that domestic wells and small local water systems, which are more common in rural areas, are not subject to the same rigorous federal regulations as larger urban water systems. This lack of regulation, combined with limited access to healthcare in these communities, further compounds the health risks associated with PFAS exposure.

A recent study [22] assessed PFAS contamination in drinking water in New Jersey (NJ), which was the first state in the U.S. to establish regulatory standards for PFASs. The researchers examined the extent of contamination and evaluated whether PFAS concentrations varied according to community sociodemographic characteristics. Black, Hispanic, and Asian communities were disproportionately affected by PFAS contamination in drinking water. The study also revealed that 93% of people of color in the state received water from community water systems (CWSs) contaminated with PFASs compared to 76% of non-Hispanic white populations. Furthermore, PFAS levels in the water exceeded the proposed Maximum Contaminant Levels (MCLs) set by both NJ and the EPA. In NJ, state-specific MCLs were previously set at 14 ng/L for PFOA, 13 ng/L for PFOS, and 13 ng/L for PFNA [108]. More recently, the EPA finalized national drinking water standards in 2024, adopting even stricter MCLs of 4 ng/L for PFOA and PFOS and applying a cumulative Hazard Index approach for other PFASs, including PFNA, PFHxS, GenX, and PFBS. Mueller et al. [22] calculated the exceedances of NJ MCLs by using the highest running annual average of four consecutive quarters of results from each sample location within a CWS, which approximates the NJ Department of Environmental Protection violation criteria. They then created indicator variables that were assigned a value of 1 when PFAS levels exceeded the threshold, and 0 when they did not. CWSs with PFAS detections exceeding NJ MCLs and the EPA’s proposed MCLs were more likely to serve higher proportions of Asian, Hispanic, and Black populations compared to non-Hispanic white populations. Given the well-established links between PFAS exposure and adverse health outcomes, these findings raise important public health concerns, as they suggest that communities of color in NJ may experience disproportionate exposure and elevated risk for PFAS-related chronic diseases. The study discloses the need for policy changes, including stricter and more comprehensive regulatory standards, enhanced water quality monitoring, and increased resources in infrastructure to ensure safe drinking water for disproportionately affected communities [109].

Further PFAS monitoring data across 18 states supports these findings, showing that communities with higher levels of PFAS contamination often have more sources of exposure [12,20]. The data revealed a significant correlation between the presence of these sources and increased PFAS contamination in drinking water [20]. For example, each additional airport, military fire training area, or industrial facility near a community’s water source was associated with a 10–108% increase in PFOA levels and a 20–34% increase in PFOS levels [20]. These results point out the environmental and health inequities that disproportionately affect communities of color, particularly in areas with higher industrial activity and a greater number of PFAS sources [12,20,102].

While PFASs are widely used in various consumer and industrial products, limited research has explored how agricultural pesticides might contribute to PFAS contamination in water supplies. Researchers aimed to assess whether communities with higher percentages of people of color experience disproportionate PFAS exposure, particularly from pesticide use [21]. The study identified agricultural regions, such as the San Joaquin Valley, where high levels of PFASs were used in pesticide applications, as areas where communities reliant on groundwater were particularly vulnerable to contamination. These regions often have less rigorous water quality monitoring, which means the residents may be unaware of their exposure to PFASs. Notably, the study did not directly measure PFAS concentrations in groundwater but instead relied on estimates derived from the quantity of pesticides used in the area. To estimate PFAS contamination near water supply wells, the study utilized data from the California Department of Pesticide Regulation, demographic information from the U.S. Census, and the locations of water supply wells. The researchers calculated pesticide use density in surrounding areas and employed spatial regression models to analyze the relationship between pesticide-related PFAS exposure and community demographics. The results indicated a significant correlation between the proportion of Latinx individuals in a community and the amount of PFASs used in nearby agricultural activities, suggesting that communities with higher Latinx populations were more likely to have PFAS-contaminated water supplies. Notably, this relationship was not directly linked to poverty levels. The analysis further revealed that after adjusting for various factors, poverty and rental housing were no longer significantly correlated with PFAS use, suggesting that SES may not be the primary driver of exposure. Instead, the study concluded that the observed racial disparities in PFAS contamination were likely driven by systemic racism—an influential yet difficult-to-quantify factor that plays a critical role in shaping environmental risks and access to resources. Between 2019 and 2021, the study estimated that approximately 3 kg of PFASs were applied near water supply wells, with 99.9% of this amount consisting of PFOS. These findings indicate the need for further research on the role of pesticides in PFAS contamination, especially in rural areas and communities of color, and highlight the importance of addressing the systemic factors contributing to environmental inequities. They also call for more comprehensive monitoring of water supplies, particularly in agricultural regions, to better protect vulnerable populations from the adverse health effects of PFAS exposure.

While PFAS-specific racial disparities remain underreported, there is a well-documented history of environmental injustice disproportionately affecting marginalized communities. For example, in the U.S., low-income communities and communities of color experience disproportionate exposure to a range of environmental hazards [110,111,112]. These include hazardous waste sites, substandard housing conditions, occupational exposures, and proximity to polluting industries. Such environmental burdens are not distributed randomly, but rather are concentrated in socioeconomically disadvantaged and racially marginalized populations, contributing to persistent health disparities across multiple outcomes. From a public health perspective, these inequities have been linked to elevated risks of chronic diseases, including neurodevelopmental disorders. Children are particularly vulnerable due to their developmental susceptibility and greater physiological sensitivity to environmental toxins. A growing body of evidence has demonstrated that early-life exposure to environmental contaminants can lead to long-term health consequences, including cognitive impairments such as reduced IQ, as seen with exposures to neurotoxicants like PFASs, polychlorinated biphenyls, tetrachloroethylene, organophosphate esters, and lead [110,113,114,115,116].

In tribal communities throughout the U.S., responses to environmental contamination are frequently delayed or less robust compared to those implemented in nontribal regions. Indigenous populations face disproportionate exposure to environmental pollutants due to the geographic locations of their communities and the cultural practices that involve close interaction with the natural environment [117,118]. Federal and state policies frequently facilitate access for extractive industries and polluting operations to tribal lands, contributing to these exposures. The legal frameworks governing tribal lands differ from those applied to nontribal lands, often resulting in regulatory inconsistencies and systemic inequities [119]. These historical and ongoing experiences have led some Indigenous communities to limit participation in research, while others engage selectively in studies that ensure equitable, collaborative partnerships. Mok et al. [119] recommend amending the UCMR5 to allocate resources and technical support aimed at increasing the inclusion of tribal public water systems. They further advocate for the U.S. EPA to facilitate the testing of additional tribal water sources, including private wells. The structure of modern environmental law in North America, which emphasizes federal–state relationships, has historically failed to adequately address pollution and environmental degradation affecting Native American lands. Complementary efforts such as public education, exposure mitigation, and environmental remediation should also be prioritized [119,120]

Importantly, health outcomes resulting from environmental injustice are preventable, as they are driven by modifiable human activities that create and perpetuate environmental degradation. Addressing these upstream drivers of exposure through policy reform, regulation, and community-driven interventions represents a critical public health priority to reduce preventable disease and promote health equity.

## 5. Intersection of PFAS Contamination and Mental Health in Communities of Color

### 5.1. Current Research

Historically, studies have focused on the physical harms caused by PFASs, but emerging evidence uncovers the importance of examining their psychological and neurodevelopmental effects. Environmental contamination, when combined with systemic inequities, may intensify mental health challenges in these populations, highlighting the need for deeper investigation into these intersecting factors.

Several longitudinal cohort studies have explored the relationship between PFAS exposure, stress, and mental health outcomes, particularly during pregnancy. One pivotal study by Eick et al. [121] laid the foundation for the Environmental Influences on Child Health Outcomes (ECHO.CA.IL) cohort. This initiative, part of the National Institutes of Health (NIH) ECHO program, merges two longitudinal pregnancy-child cohorts—Illinois Kids Development Study (IKIDS) and Chemicals in Our Bodies (CIOB)—to create a more diverse sample and address limitations of smaller, less inclusive studies. The ECHO.CA.IL cohort includes participants from various geographic, socioeconomic, racial, and ethnic backgrounds, enabling a comprehensive investigation into the effects of endocrine-disrupting chemicals (EDCs) like PFASs on prenatal stress, birth outcomes, and cognitive development in infants. The study found that younger women, high-school graduates, and women from racial and ethnic minority groups experienced higher levels of stressful life events and perceived stress compared to white women. Notably, racial and ethnic minority groups were associated with lower birth weight z-scores compared to white women, and socioeconomic factors, including unmarried status, were also linked to reduced birth weight. Although no individual PFAS compound was identified as a significant contributor, the study observed an association between combined PFASs and stress mixtures and reduced birth weight. Eick et al. [122] conducted another study using the ECHO.CA.IL cohort to examine both the individual and combined effects of chemical and non-chemical stressors on birth outcomes and neurodevelopment during infancy and early childhood. This study was among the first to apply environmental mixture methods to jointly assess chemical and non-chemical stress exposures. The authors found that prenatal exposure to PFASs, perceived stress, and depression, when modeled together as a mixture, was modestly associated with lower birthweight z-scores. The combined effects of PFASs and psychosocial stressors were greater than those observed for individual exposures alone. The mixture analysis suggested that both PFAS exposure and stress responses contribute to fetal growth outcomes. The authors recommend that future research should investigate whether non-chemical stressors may enhance susceptibility to environmental chemical exposures.

Further analysis by Eick et al. [123] focused on pregnant African American women in Atlanta, investigating the combined effects of PFAS exposure and psychosocial stress—such as perceived stress, depression, anxiety, and gendered racial stress—on birth outcomes. The study found that co-exposure to PFASs and psychosocial stressors during pregnancy was associated with lower birth weight for gestational age, emphasizing the compounded impact of environmental and social stressors on maternal and fetal health.

Schildroth et al. [124] examined the relationship between PFAS exposure and perceived stress among reproductive-aged women in Detroit, Michigan, as part of the Study of Environment, Lifestyle, and Fibroids (SELF) cohort. Although the overall association between PFASs and perceived stress was modest, PFOA demonstrated the strongest association. These findings indicate that while PFAS-related psychological effects may not be uniformly observed across all populations, individuals exposed to compounded socioeconomic stressors—such as immigrant or economically marginalized communities—may exhibit heightened vulnerability to these effects. Consistent with this, the study reported that perceived stress linked to PFAS exposure was greatest among women residing in economically disadvantaged areas [124]. Age is an additional factor influencing susceptibility, as children and adolescents are particularly sensitive due to critical developmental windows and the potential for cumulative exposure beginning in early life.

Moreover, the cross-sectional study by Eick et al. [125] further explored the relationship between PFASs and the body’s stress response, specifically through the measurement of corticotropin-releasing hormone (CRH) during pregnancy. The study found a strong positive correlation between PFNA and CRH levels, particularly among economically disadvantaged women facing food insecurity and financial strain. This suggests that PFAS exposure may exacerbate stress responses in vulnerable populations, leading to potential long-term health consequences.

The mental health implications of PFAS exposure have also been explored through qualitative research. A study by Calloway et al. [13] investigated the lived experiences of residents in six PFAS-contaminated U.S. cities. Participants reported heightened stress and anxiety stemming from uncertainty, lack of control over environmental risks, and erosion of trust in governmental institutions. Concerns about health, financial strain, and emotional distress illustrated the layered mental health burden experienced by affected communities.

International findings echo these concerns. A cross-sectional study [126] in Australia found elevated self-reported anxiety levels among individuals living in PFAS-exposed communities, although associations with serum PFAS concentrations were less clear. This adds to the growing evidence linking environmental contamination with adverse mental health outcomes.

Lastly, the study conducted by Bashir and Obeng-Gyasi [127] assessed PFAS exposure and allostatic load (AL)—an index of chronic physiological stress—among a diverse cohort in North Carolina. Non-Hispanic Black participants exhibited higher AL levels, which correlated positively with PFAS exposure, particularly PFOA. These findings indicate that PFASs may contribute to heightened chronic stress and exacerbate existing mental health disparities in marginalized populations.

While the physiological effects of PFAS exposure remain the primary focus of most research, growing evidence points to significant mental health consequences—particularly for communities of color already burdened by systemic inequities. Studies [13,121,122,123,124,125,126,127] reviewed here reveal how PFAS exposure can intersect with socioeconomic stressors to compound psychological distress, affect birth outcomes, and elevate chronic stress markers. These findings emphasize the urgent need for expanded research agendas and targeted public health policies that address both the physical and mental health dimensions of PFAS exposure in vulnerable populations.

### 5.2. Challenges in Understanding Mental Health Impacts

As concerns surrounding PFAS contamination continue to rise, a growing body of research has linked these substances to a variety of physiological disorders in humans [17,89,95,96,126]. However, the connection between PFAS exposure and psychological disorders remains underexplored, despite emerging evidence suggesting potential associations with mental health issues and their consequential physiological effects, such as altered birthweight. Although previous studies provide valuable insights into the complex relationship between PFASs and mental health, they also highlight significant gaps in the current literature, particularly with respect to demographic sensitivities and exposure patterns across different populations.

A key limitation in the current body of research lies in the measurement tools used to assess the relationship between environmental PFAS exposure and psychological stress. Instruments such as the Perceived Stress Scale (PSS), and depression scales like the CES-D (Center for Epidemiologic Studies Depression Scale) and EPDS (Edinburgh Postnatal Depression Scale), are widely used to assess stress and mental health outcomes. However, these tools predominantly rely on self-reported data, which are subject to various biases, including recall bias, social desirability bias, and individual differences in the perception of stress. This reliance on self-reporting may obscure the true extent of the mental health impacts associated with PFAS exposure and fail to capture the nuanced experiences of individuals in different demographic groups.

Further complicating the matter is the challenge of establishing causal links between PFAS exposure and psychological stress. While studies suggest correlations between PFAS exposure and stress regulation in the body, the causal mechanisms underlying these associations remain unclear [121,122,123,125]. The cross-sectional nature of many studies, which often capture data at a single point in time, limits the ability to infer causality. Longitudinal studies, by contrast, would allow for a more comprehensive understanding of how PFAS exposure accumulates over time and how it interacts with other factors (e.g., socioeconomic stressors, access to healthcare, and environmental conditions) to influence mental health outcomes.

Targeted studies focusing on communities of color remain limited, despite their importance in capturing distinct exposure patterns and susceptibilities shaped by structural and systemic inequities [12,14,41,104,123]. Without such research, the field risks neglecting critical variations in PFAS exposure and associated mental health outcomes across racial, ethnic, and socioeconomic groups, thereby hindering a comprehensive understanding of population-level impacts.

While the scientific literature on PFAS exposure and mental health is expanding, several limitations remain in the current body of research. The reliance on self-reported data, the predominance of cross-sectional studies, and the lack of focus on specific demographic groups hinder our ability to fully understand the psychological consequences of PFAS exposure. Future research should prioritize longitudinal studies that can assess long-term exposure and its cumulative effects on mental health, as well as targeted studies that explore the impact of PFASs on diverse communities. These efforts are essential for developing more effective public health policies and interventions aimed at mitigating the mental health and physiological risks associated with PFAS contamination.

### 5.3. Cultural and Societal Factors

Researchers [121,128] reported that PFAS exposure levels were generally higher among women with higher educational attainment, those who were married or living with a partner, and those with one or more previous births. In contrast, PFAS concentrations were lower among Hispanic and Black women in the same study. These findings differ from those of Bashir and Obeng-Gyasi [127], who reported elevated PFAS exposure among pregnant women with lower educational attainment and from non-Hispanic Black backgrounds. The discrepancies between these studies may be influenced by a range of cultural, societal, and demographic factors, particularly within underserved communities. In many minority and low-income populations, the perception and reporting of mental health issues are often shaped by cultural norms and societal expectations. For instance, mental health concerns in racial minority groups, such as Black and Hispanic populations, tend to be more stigmatized compared to other groups [129,130,131,132]. Black women from lower socioeconomic backgrounds report higher levels of stigma related to mental health, which may result in lower utilization of mental health services [131,133]. This phenomenon can be understood within the broader context of historical and ongoing social inequities, such as migration experiences, gender-based discrimination, and mistrust of medical institutions due to systemic racism and past abuses [131].

Such stigmas, compounded by environmental racism, may exacerbate psychological distress within these communities. The disproportionate exposure to environmental hazards, including PFASs, in underserved populations contributes to heightened feelings of vulnerability and fear [129]. As environmental risks disproportionately affect minority communities, these populations may experience increased anxiety and stress, which can have significant implications for mental health outcomes [129,134,135]. These societal and structural factors provide a critical framework for interpreting existing PFAS data and highlight the need for more inclusive, culturally sensitive research that considers the unique experiences of communities of color [136].

Future research should consider the socio-cultural context in which PFAS exposure occurs and its potential psychological impact on marginalized communities. This would involve accounting for variables such as stigma, trust in healthcare systems, and the intersectionality of race, gender, and SES. Addressing these factors is essential to improving the quality of research and ensuring that future studies are better equipped to support vulnerable communities in their fight for environmental justice and better health outcomes.

### 5.4. Limitations

Several important limitations should be considered when interpreting associations between PFAS exposure and mental health outcomes. Although numerous studies have reported associations between PFAS exposure and symptoms commonly observed in mental health disorders, these findings do not establish direct causal relationships with clinically diagnosed conditions. Much of the existing literature relies on self-reported symptom measures, which, while validated for screening purposes, broadly capture psychological distress that may reflect multiple underlying etiologies. In addition, these associations are influenced by complex sociodemographic, environmental, and psychosocial factors, including socioeconomic status, racial and ethnic disparities, historical trauma, and environmental injustice, all of which may confound or modify the observed relationships between PFAS exposure and mental health vulnerability. Few studies to date have incorporated objective physiological measures such as neuroendocrine biomarkers, indicators of systemic inflammation, or neuroimaging assessments, which are necessary to elucidate potential mechanistic pathways. Therefore, the current findings should be interpreted with caution. Future research employing longitudinal designs, comprehensive confounder adjustment, and the integration of biological markers is needed to better characterize the mental health effects of PFAS exposure.

## 6. Policy and Regulatory Landscape

### 6.1. Current PFAS Regulations

#### 6.1.1. International Regulations

International comparisons of PFAS exposure data are complicated by numerous methodological, regulatory, and logistical challenges that limit direct interpretation and synthesis. First, significant heterogeneity exists in the selection of PFAS analytes, laboratory methodologies, and detection sensitivities employed across studies and surveillance programs. While certain countries focus primarily on legacy compounds such as PFOSs and PFOAs, others incorporate emerging replacement chemicals like GenX or various short-chain PFASs, resulting in datasets that are often non-equivalent or only partially overlapping. Second, differences in biomonitoring study designs—such as variation in participant demographics, biological matrices sampled (e.g., serum, urine, or breast milk), and timing of specimen collection—introduce additional variability unrelated to actual exposure levels, thereby complicating cross-country comparisons in the absence of standardized protocols. Third, national regulatory frameworks differ in both scope and stringency, shaping which PFAS compounds are prioritized, how exposure data are reported, and the intervals at which monitoring occurs. For example, some countries utilize aggregate hazard indices to estimate cumulative risk from multiple PFASs, while others report individual compound concentrations without integrated risk assessments, limiting the comparability of exposure metrics across jurisdictions. Furthermore, disparities in data availability and transparency pose additional barriers: while some nations support open-access databases, others restrict data dissemination due to privacy protections or policy constraints, impeding comprehensive global assessments and cross-national research collaborations. Addressing these complexities requires sustained international coordination to standardize analytical approaches, target analyte panels, and reporting standards.

In Europe, the European Chemicals Agency (ECHA) has actively pursued stringent measures to restrict and phase out the use of certain PFAS products. Under the EU’s Persistent Organic Pollutants (POPs) Regulation, PFOS has been banned for over a decade, and the EU continues to make rapid strides toward further restrictions. A proposed EU-wide ban on thousands of PFAS chemicals is under consideration, with a final agreement expected by 2025 [137].

Similarly, Canada has implemented some controls on PFAS chemicals through the Canadian Environmental Protection Act (CEPA) and the Chemicals Management Plan (CMP), which include limitations on the production, importation, and distribution of certain PFAS compounds. However, Canada has yet to set national restrictions for PFASs across all sectors [138].

In Australia and New Zealand, the PFAS National Environmental Management Plan (PFAS NEMP) has established guidelines for PFAS contamination in soil and groundwater, with ongoing efforts to address waste management and remediation. This initiative represents a concerted effort to mitigate PFAS pollution in the country [139].

In Asia, countries such as Japan, South Korea, and China have introduced regulations on PFASs, but comprehensive national bans have not yet been implemented [65]. Although China is a major global supplier of chemicals, the country has not made significant changes to its PFAS regulations since 2023. Given China’s dominant position in the global chemical supply chain, its regulatory decisions regarding PFASs will likely have far-reaching implications for global environmental health.

#### 6.1.2. U.S. Regulations

Table 1 summarizes key federal, state, academic, and non-governmental organizations (NGOs) involved in PFAS research, regulation, monitoring, and advocacy in the U.S. The organizations and initiatives were identified through a targeted review of the peer-reviewed literature, U.S. government agency reports (e.g., EPA, CDC, and NIEHS), public databases, and policy documents published between 2020 and 2024. Relevant state-level regulatory bodies and prominent NGOs were included based on their documented engagement in PFAS-related activities such as monitoring, remediation, public health surveillance, legal advocacy, and community-based research. This comprehensive review reflects the multi-sectoral and collaborative approach necessary to address the complex environmental and public health challenges posed by PFAS contamination.

In the U.S., regulatory efforts addressing PFASs have primarily focused on drinking water, with soil contamination largely addressed indirectly through groundwater regulation [140]. The estimated cost of mitigating PFASs in drinking water is projected to range from USD 37.1 to USD 48.3 billion over the next five years, with annualized operations and maintenance costs estimated at USD 2.7 to USD 3.5 billion [141]. These substantial financial burdens may partially explain the limited national attention toward PFAS contamination in soil.

Although states such as Delaware and Virginia have initiated soil-specific PFAS regulations, the absence of consistent federal standards heightens risks to agricultural systems and food safety [142]. On 10 April 2024, the EPA finalized the first National Primary Drinking Water Regulation (NPDWR) [143] for PFASs, establishing enforceable MCLs for six compounds—PFOA, PFOS, PFHxS, PFNA, and HFPO-DA—as well as a cumulative Hazard Index MCL for mixtures of PFHxS, PFNA, HFPO-DA, and PFBS. Public water systems must begin monitoring and reporting PFAS concentrations by 2027 and implement treatment and public notification protocols by 2029 if MCLs are exceeded. This regulation is backed by nearly USD 1 billion in Bipartisan Infrastructure Law funding, contributing to a USD 9 billion national effort to address PFASs and other emerging contaminants, with an additional USD 12 billion allocated to broader drinking water infrastructure upgrades. While this regulatory framework is anticipated to reduce PFAS exposure for approximately 100 million people, concerns have emerged about inequities in the distribution of remediation resources [143].

Soil contaminated with PFASs and other persistent pollutants is linked to oxidative stress, inflammation, and epigenetic modifications that contribute to a range of chronic conditions, including cancer, neurodegenerative disorders, cardiometabolic disease, myocardial infarction, and hypertensive complications in pregnancy [144]. Agricultural workers remain especially vulnerable, with millions affected annually by chemical exposures. Yet, no federal standards currently regulate PFAS concentrations in soil, highlighting an urgent gap in environmental and public health protection.

Populations disproportionately burdened by PFAS exposure, often with limited access to remediation and healthcare resources, warrant focused intervention. As scientific evidence linking PFASs to adverse health outcomes continues to expand, it is imperative that policymakers adopt inclusive, evidence-based regulatory frameworks that prioritize exposure reduction in marginalized communities and bridge persistent gaps in both research and policy implementation.

### 6.2. Proposed Recommendations

**Figure 6:**
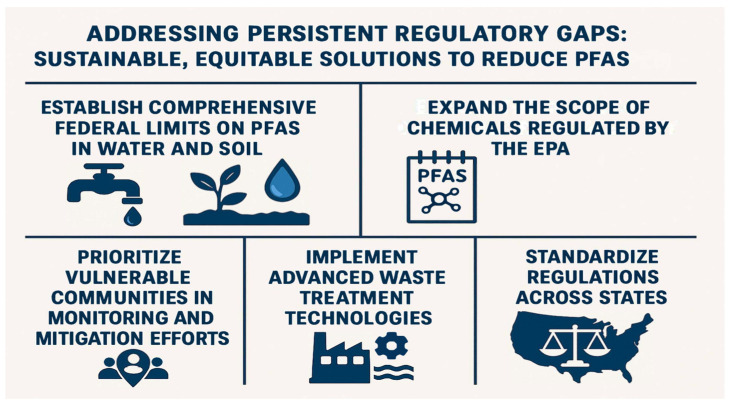
Regulatory framework addressing per- and polyfluoroalkyl substances exposure with equity-focused recommendations.

To address persistent regulatory gaps and promote sustainable, equitable solutions, the following recommendations (Figure 6) focus on reducing PFAS exposure, particularly in marginalized communities: (1) Establish Comprehensive Federal Limits on PFASs in Water and Soil—Current federal regulations primarily target PFASs in drinking water, while soil contamination remains largely unregulated. As PFASs in soil can enter the food chain and pose risks to agricultural productivity and food safety, it is essential to establish enforceable federal standards for PFAS levels in both water and soil. A nationwide study found PFASs at nearly every soil site tested, emphasizing its role as a major environmental reservoir [145]. Uniform federal limits would provide consistent protections and prevent cross-state disparities in public health safeguards. (2) Expand the Scope of PFAS Chemicals Regulated by the EPA—The EPA currently regulates only a small subset of PFASs, leaving thousands of structurally similar compounds with comparable toxicity unaddressed. Expanding the regulatory framework to include a broader range of PFASs would reduce the likelihood of industry substitution with equally hazardous alternatives, thereby mitigating long-term health and environmental risks. (3) Prioritize Vulnerable Communities in Monitoring and Mitigation Efforts—Research indicates that PFAS contamination disproportionately affects low-income and racially marginalized populations [20]. These communities should be prioritized in research, regulatory efforts, and federal resource allocation. Strategies must include enhanced exposure monitoring, access to treatment technologies, and support for community-led remediation efforts. Empowering local communities through education and involvement in regulatory decision-making is essential to advancing environmental justice. (4) Implement Advanced Waste Treatment Technologies—Conventional PFAS disposal methods, such as incineration and landfilling, are inadequate and risk further environmental contamination. Technologies like supercritical water oxidation (SCWO) offer more effective alternatives by breaking down PFASs into inert byproducts. Integrating SCWO into waste management systems can significantly reduce environmental burdens and limit recontamination [146]; (5) Standardize Regulations Across States—State-level PFAS regulations vary widely, creating a fragmented system of protection. As of 2025, 37 states have proposed PFAS-related legislation, with at least 19 advancing these bills and two—New Mexico and Virginia—enacting them into law [147]. These efforts largely focus on consumer products, AFFF, biosolids, pesticides, and water systems. Federal intervention is necessary to establish a coherent regulatory baseline across all states to ensure uniform enforcement and equitable protection.

A coordinated approach involving federal regulation, technological innovation, community prioritization, and standardized state policies is essential for effectively mitigating PFAS-related health risks. These steps will contribute to more equitable and sustainable outcomes, particularly for communities most at risk.

### 6.3. Community Advocacy and Solutions

Community-led initiatives have played a critical role in raising awareness and advancing local PFAS remediation efforts. PFAS-REACH (Research, Education, and Action for Community Health), a five-year National Institute of Environmental Health Sciences (NIEHS)-funded project, exemplifies how collaborative research can inform public health responses through the integration of scientific investigation and community engagement [27]. Similarly, Silent Spring Institute [148] has partnered with Cape Cod residents for over two decades to assess PFAS impacts and monitor water quality.

In the U.S., grassroots efforts like the Alliance for a Healthy Tomorrow [149] have advocated for bans on PFASs in firefighter personal protective equipment (PPE) and pushed for greater corporate accountability. The NIEHS-funded STEEP (Sources, Transport, Exposure, and Effects of Per- and Polyfluoroalkyl Substances) Center [150] supports interdisciplinary research to elucidate exposure–disease linkages, complementing these advocacy efforts.

Across Europe and Asia, environmental organizations have driven regulatory reforms. The environmental group BUND (Bund für Umwelt und Naturschutz Deutschland) [151] in Germany successfully lobbied for a PFAS phase-out by 2030, while the Rotterdam court’s ruling against Chemours for contamination in the Netherlands marked a legal milestone [152]. In South Korea, the KFEM (Korean Federation for Environmental Movement) [153] has worked to curb PFAS emissions from military bases, supported by the government’s 2019 Stockholm Convention implementation plan.

Community-academic partnerships are also evident in Maine, where the Micmac Nation is collaborating with researchers to use fiber hemp for soil remediation on a contaminated former air force base [154]. In Ridgewood, NJ, municipal leadership, guided by community input, developed a PFAS Treatment Master Plan approved in 2021. This initiative consolidated 31 treatment facilities into 12 sites using granular activated carbon to reduce PFASs to non-detectable levels [155].

These examples highlight the transformative potential of grassroots initiatives in shaping PFAS policy, research, and remediation. Community involvement remains vital in bridging regulatory gaps and promoting environmental justice.

### 6.4. Community Engagement

Effective advocacy relies on public awareness, and the use of media and digital tools enhances community outreach. The EPA’s PFAS Communications Toolkit equips organizations with educational resources to inform the public [156]. Campaigns like “Fight Forever Chemicals”, launched alongside Dark Waters, demonstrate how storytelling and media can galvanize public action [157].

Mapping projects from Northeastern University’s Social Science Environmental Health Research Institute [158] and the Environmental Working Group (EWG) [159] have visualized PFAS contamination nationwide, helping to mobilize community responses and policy changes.

Advancing equitable policymaking necessitates the integration of participatory frameworks that meaningfully involve impacted communities in the formulation and implementation of environmental policies, ensuring that decisions reflect communi-ty-informed insights and localized knowledge. Reflecting this approach, the U.S. De-partment of the Air Force recently announced two major initiatives aimed at accelerating the remediation of PFAS contamination at the former Wurtsmith Air Force Base in Iosco County, Michigan [160]. These efforts are being carried out in collaboration with the Michigan Department of Environment, Great Lakes, and Energy, alongside other local governmental and community stakeholders, demonstrating the importance of mul-ti-level, community-engaged response strategies [160]. The National PFAS Contamination Coalition [161] emphasizes the need for including affected populations in decision-making. Developing culturally tailored resources and collaborating with trusted local organizations can ensure that outreach efforts resonate with diverse audiences and promote community resilience.

## 7. Conclusions

The PFAS crisis demands a multidimensional response that integrates regulatory action, scientific research, and grassroots advocacy. Community-led efforts, both domestically and internationally, have proven essential in raising awareness and catalyzing policy shifts, yet significant disparities remain—particularly in recognizing and addressing the mental health and psychosocial burdens experienced by disproportionately affected populations, including communities of color. To move beyond fragmented interventions, future research must prioritize community-based participatory approaches that amplify local knowledge, enhance trust, and ensure the contextual relevance of findings.

Incorporating mental health dimensions into environmental health studies is critical for capturing the full scope of PFAS-related harm. Investigations into the neurodevelopmental and neurological consequences of PFAS exposure—such as emerging links to ADHD and Parkinson’s disease—emphasize the need for a more integrative research paradigm. Concurrently, U.S. regulatory progress, while encouraging, remains incomplete; federal action must expand beyond drinking water standards to include soil remediation, agricultural safety, and broader exposure pathways by establishing enforceable, nationwide MCLs for key PFAS compounds; mandating the industry disclosure and phase-out of PFASs in consumer products; investing in the clean-up of legacy contamination sites; and prioritizing community involvement and funding for vulnerable communities.

Ultimately, achieving environmental justice in the context of PFAS contamination requires a sustained, inclusive strategy that bridges science, policy, and community engagement. By elevating the voices of those most affected and advancing interdisciplinary research, we can develop more equitable and effective solutions to one of the most pressing environmental health challenges of our time.

## Figures and Tables

**Table 1:** Major organizations and initiatives addressing per- and polyfluorinated substances in the United States.

Organization/Agency	Key PFAS Roles and Activities	Type
**Environmental Protection Agency**	PFAS Strategic Roadmap; drinking water regulations; Superfund site designations; national testing strategy; risk assessments	Federal Regulator
**Agency for Toxic Substances and Disease Registry**	PFAS Exposure Assessment Studies (PEAS); Multi-Site Health Study; toxicological profiles	Federal Public Health
**Centers for Disease Control and Prevention**	Biomonitoring through NHANES; collaboration with ATSDR on health studies	Federal Public Health
**Department of Defense**	Remediation of PFASs at military installations; research on AFFF contamination	Federal Defense
**Food and Drug Administration**	Surveillance of PFASs in food, food packaging, and the food supply chain	Federal Regulator
**National Institute of Environmental Health Sciences**	Superfund Research Program (SRP); PFAS toxicity studies; grant funding	Federal Research
**U.S. Geological Survey**	Environmental monitoring and mapping of PFASs in water, soil, and wildlife	Federal Scientific Agency
**White House Council on Environmental Quality**	Interagency PFAS Council to coordinate federal PFAS activities	Federal Executive
**State-Level Agencies**	Independent PFAS regulations, health advisories, and site monitoring	State Regulators
**Environmental Working Group**	PFAS mapping; public education; policy advocacy	NGO
**Natural Resources Defense Council**	Legal and policy advocacy; PFAS litigation	NGO
**Safer States**	State-level PFAS regulation tracking; coalition building	NGO
**Green Science Policy Institute**	Research and advocacy for eliminating PFASs in products	NGO
**National Academies of Sciences, Engineering, and Medicine**	Scientific reports; guidance for PFAS health monitoring	Academia
**Academic PFAS Consortia**	PFAS research; NIEHS-funded Superfund centers; health outcome studies	Academia

## Data Availability

Not applicable.

## Abbreviations

The following abbreviations are used in this manuscript:

ADHD	attention-deficit hyperactivity disorder
AFB	Air Force Base
AFFF	aqueous film-forming foam
AL	allostatic load
ATP	adenosine triphosphate
ATSDR	Agency for Toxic Substances and Disease Registry
BBB	blood–brain barrier
BUND	Bund für Umwelt und Naturschutz Deutschland
CDC	Centers for Disease Control and Prevention
CEPA	Canadian Environmental Protection Act
CES-D	Center for Epidemiologic Studies Depression Scale
CIOB	Chemicals in Our Bodies
9CLPF	9-chlorohexadecafluoro-3-oxanonane-1-sulfonic acid
CMP	Chemicals Management Plan
CNS	central nervous system
CRH	corticotropin-releasing hormone
CTRL	control
CWS	community water systems
DAT	dopamine transporter
DP1	neuronal commitment phase
DP2	neuronal precursor phase
DP3	mature dopaminergic differentiation phase
ECHA	European Chemicals Agency
ECHO.CA.IL	Environmental Influences on Child Health Outcomes
EDCs	endocrine-disrupting chemicals
EGLE	Environment, Great Lakes, and Energy
eGFR	estimated glomerular filtration
EPA	Environmental Protection Agency
EPDS	Edinburgh Postnatal Scale
EWG	Environmental Working Group
FDA	Food and Drug Administration
HFPO-DA (Gen X^®^)	hexafluoropropylene oxide dimer acid
IARC	International Agency for Research on Cancer
IKIDS	Illinois Kids Development Study
KFEM	Korean Federation for Environmental Movement
MCLs	Maximum Contaminant Levels
MoCRA	Modernization of Cosmetics Regulation Act of 2022
NEMP	National Environmental Management Plan
NF-H	neurofilament heavy chain
NHANES	National Health and Nutrition Examination Survey
NIEHS	National Institute of Environmental Health Sciences
NIH	National Institutes of Health
NGOs	non-governmental organizations
NJ	New Jersey
NPDWR	National Primary Drinking Water Regulation
NY	New York
PFAS	per- and polyfluoroalkyl substances
PFBA	perfluorobutanoic acid
PFBS	perfluorobutane sulfonate
PFDA	perfluorodecanoic acid
PFHpA	perfluoroheptanoic acid
PFHxS	perfluorohexane sulfonate
PFNA	perfluorononanoic acid
PFOA	perfluorooctanoic acid
PFOS	perfluorooctane sulfonic acid
PFOSA	perfluorooctane sulfonamide
POPs	persistent organic pollutants
PPE	personal protective equipment
PPAR	peroxisome proliferator-activated receptor
PSS	Perceived Stress Scale
REACH	Research, Education, and Action for Community Health
ROS	reactive oxygen species
SCWO	supercritical water oxidation
SELF	Study of Environment, Lifestyle, and Fibroids
SES	socioeconomic status
STEEP	Sources, Transport, Exposure, and Effects of Per- and Polyfluoroalkyl Sub-stances
TH	tyrosine hydroxylase
UCMR 5	Fifth Unregulated Contaminant Monitoring Rule
US	United States
